# Mutant phenotype analysis suggests potential roles for C-type natriuretic peptide receptor (NPR-B) in male mouse fertility

**DOI:** 10.1186/1477-7827-12-64

**Published:** 2014-07-10

**Authors:** Chizuru Sogawa, Yasuhiro Fujiwara, Satoshi Tsukamoto, Yuka Ishida, Yukie Yoshii, Takako Furukawa, Tetsuo Kunieda, Tsuneo Saga

**Affiliations:** 1 Molecular Imaging Center, National Institute of Radiological Sciences, 4-9-1 Anagawa, Inage-ku, Chiba-shi, Chiba 263-8555, Japan; 2 The Jackson Laboratory, 600 Main Street, Bar Harbor, ME 04609, USA; 3 Graduate School of Environmental and Life Science, Okayama University, 1-1-1 Tsushima-naka, Kita-ku, Okayama-shi, Okayama 700-8530, Japan; 4 Laboratory Animal and Genome Sciences Section, National Institute of Radiological Sciences, 4-9-1 Anagawa, Inage-ku, Chiba-shi, Chiba 263-8555, Japan

**Keywords:** CNP, NPR-B, cGMP, SLW mouse, Smooth muscle, Paraphimosis, Infertility, Spermatogenesis

## Abstract

**Background:**

C-type natriuretic peptide (CNP) signaling through its receptor natriuretic peptide receptor B (NPR-B) is a key molecule for mammalian reproduction, and known to play important roles in female fertility. However, the function of these peptides in mouse male reproduction remains largely unknown. To determine the role of CNP/NPR-B signaling in male reproduction we investigated phenotype of *Npr2*-deficient short-limbed-dwarfism (*Npr2*^*slw*/*slw*^) mice, which have been shown to have gastrointestinal (GI) abnormalities.

**Findings:**

In homozygous *Npr2*^*slw*/*slw*^ mice, spermatogenesis is developmentally delayed at both 2 and 4 weeks of age, with vacuolation and degenerating apoptotic germ cells being observed at 3 weeks age. However, the adult *Npr2*^*slw*/*slw*^ mice exhibited apparently normal spermatogenesis, albeit with some aberrant spermatids, suggesting that developmental delay was overcome. In addition, the adult *Npr2*^*slw*/*slw*^ mice showed abnormal penile morphology (paraphimosis).

**Conclusions:**

The potential role of CNP signaling via the NPR-B receptor in male fertility appears to be mediated not through germ-cell development, but may be through maintenance of normal penile function.

## Findings

### Background

The mouse *Npr2* gene encodes natriuretic peptide receptor B, NPR-B (also known as NPR2), which is a known receptor for C-type natriuretic peptide, CNP (encoded by the *Nppc* gene, and also known as NPPC). NPR-B synthesizes intracellular cyclic guanosine monophosphate (cGMP) [1], which acts as a secondary messenger and is degraded by cGMP-specific phosphodiesterase type 5 (PDE5) [2]. The peptide and its receptor, CNP/NPR-B are expressed in the nervous and vascular systems [3-5], including the gastrointestinal tract [6], where they are thought to act as a local paracrine or autocrine regulators [5].

A number of reports have demonstrated an important role for CNP in reproductive regulation in females [7]. Although male *Npr2* exhibit reduced reproductive functions, it is not known why male *Npr2* mutants are infertile or sub-fertile. Most spontaneous *Npr2*^*slw/slw*^ (SLW: Short-limbed dwarfism) males fail to mate, but a limited number of these mutants generated offspring [8]. Males homozygous for another spontaneous mutation, *Npr2*^*pwe/pwe*^ (peewee mouse), were sub-fertile, but exhibited normal testis histology [9]. Interestingly, the male *Npr2*-KO mouse was infertile but exhibited normal spermatogenesis [10]. Observations on male reproduction were mentioned only briefly in these studies and not pursued in detail.

In a previous report, it was suggested that CNP/NPR-B signaling could lead to relaxation of seminiferous tubules and thereby regulate sperm transportation and testicular blood supply [11]. This signaling is also believed to modulate spermatozoa motility, testicular germ cell development and testosterone synthesis in mice [12]. In addition, it was also reported that CNP regulates blood-testis barrier (BTB) dynamics in adult rats [13]. These reports suggested that CNP is a key factor for spermatogenesis. Therefore, it might be expected that absence of NPR-B would also impact on spermatogenesis, and indeed, reduced fertility is a phenotype in several *Npr2* mutants. However, the *Npr2* mutant mice also exhibit other phenotypes that might ultimately affect fertility. For example, *Npr2*^*slw/slw*^ mutants display gastrointestinal (GI) dysfunctions and high pre-weaning death, due to lack of CNP/NPR-B signaling [6,14]. In the case of the few surviving *Npr2*^*slw/slw*^ mice, the GI disorders might cause nutritional restriction, which is known to contribute to aberrant spermatogenesis and reduced number of germ cells [15,16].

This study was undertaken to provide more complete and definitive observation using mice homozygous for a mutant allele of *Npr2*, *Npr2*^*slw*^, which could contribute to determination of a possible link between the CNP/NPR-B signaling pathway and male reproduction.

## Methods

### Mice

The *Npr2*^*slw*^ mutant line, herein referred to as SLW mice were maintained under standard conditions. An inbred SLW strain was created by a single backcross to the ddY mouse (Japan SLC, Inc., Shizuoka, Japan), and subsequent mating between heterozygous mice. In this study, the mice used as comparative controls were either heterozygotes or wild type for the non-mutated allele; collectively they are referred to as control. All animal experiments were carried out in accordance with institutional guidelines regarding animal care and handling, and the experimental protocol was approved by the Institutional Animal Care and Use Committee of the National Institute of Radiological Sciences.

### Histology and TUNEL assay

Testes were prepared and fixed in Bouin’s solution for 1 h (0 day postpartum (dpp)), 2 h (7 dpp), 4 h (14 dpp), overnight (21 dpp), and 24 h (28 dpp, 35 dpp, and adult) at room temperature (RT), and then immersed in 70% ethanol for 48 h. The specimens were dehydrated and embedded in paraffin. Subsequently, 2-μm-thick sections were cut, attached to glass slides (MAS-GP Type A; Matsunami Glass Ind., Ltd., Osaka, Japan), and then deparaffinized, rehydrated, and subjected to hematoxylin and eosin staining.

The TUNEL assay was performed for detection of apoptotic cells using ApopTag® Peroxidase *in situ* Apoptosis Detection Kit (CHEMICON® International, Inc. CA, USA) according to the manufacturer’s instructions.

### Statistical analysis

Data are expressed as the means ± SD or SE. The statistical significance of differences in mean values was assessed by Student’s t-test.

## Results

### Developmental delay of spermatogenesis in juvenile but not adult *Npr2* mutant mice

**Figure 1:**
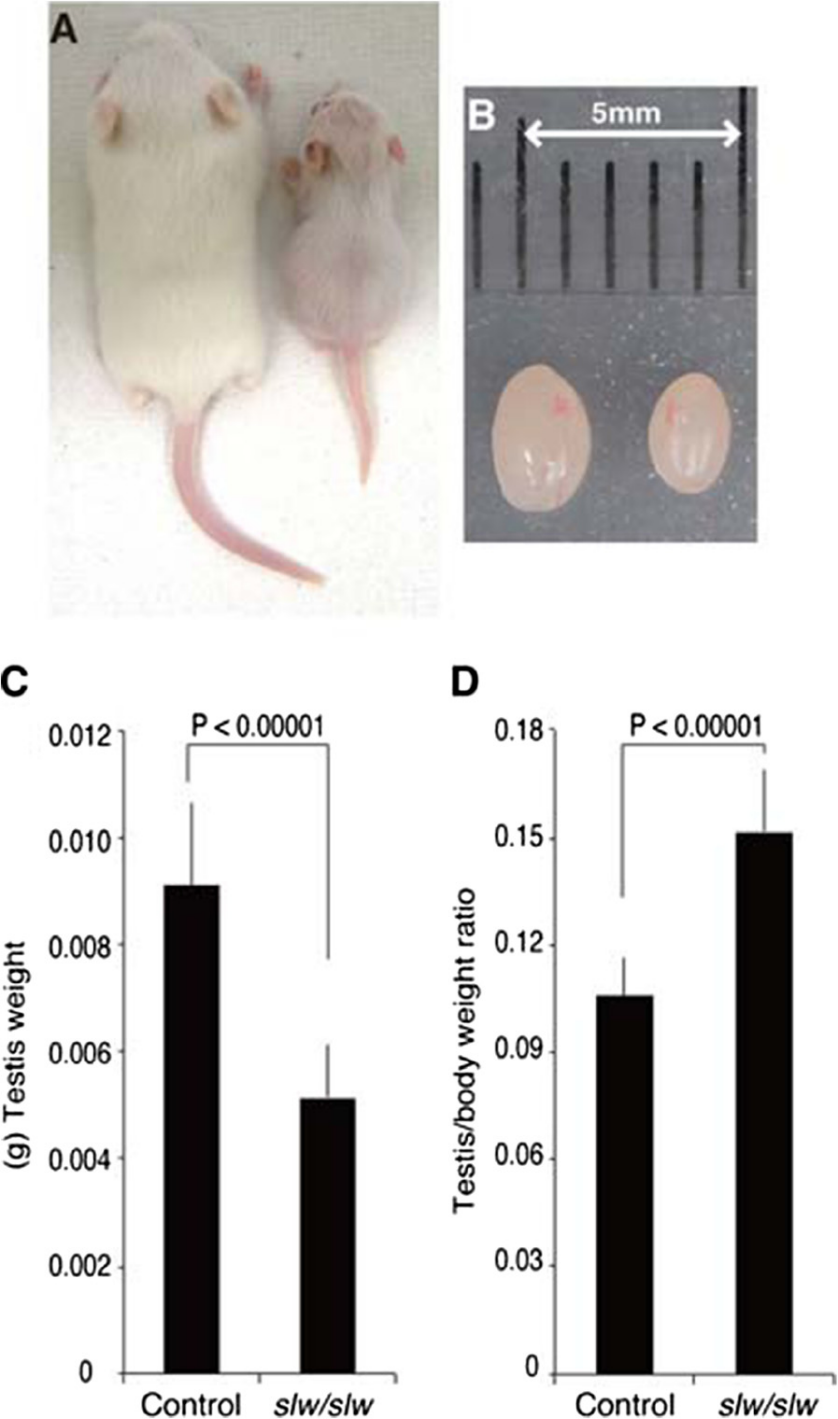
**Phenotype of testes of *Npr2***^***slw/slw ***^**mice.** The body size **(A)** and testis size **(B)** at 12 dpp of the control litter mate (left) and *Npr2*^*slw/slw*^ mutant (right). Testis weights of the control and *Npr2*^*slw/slw*^ mutants **(C)**, with testes to body weight ratio **(D)**. **C** and **D**: n = 4 mice (n = 8 testes) for both control and *Npr2*^*slw/slw*^ mice. The data shown are means (columns) and SDs (bars) **(C and D)**.

As previously reported, the *Npr2*^*slw/slw*^ mutant mice exhibited dwarfism and GI distention with gas (Figure 1A). The testes of *Npr2*^*slw/slw*^ mice were smaller than control testes (Figure 1B), with the *Npr2*^*slw/slw*^ testis weight reduced by about 50% compared to that of control (Figure 1C). However, the testis to body weight ratio of *Npr2*^*slw/slw*^ mice was significantly larger in the *Npr2*^*slw/slw*^ than in control mice, reflecting the dwarfism (Figure 1D).

**Figure 2:**
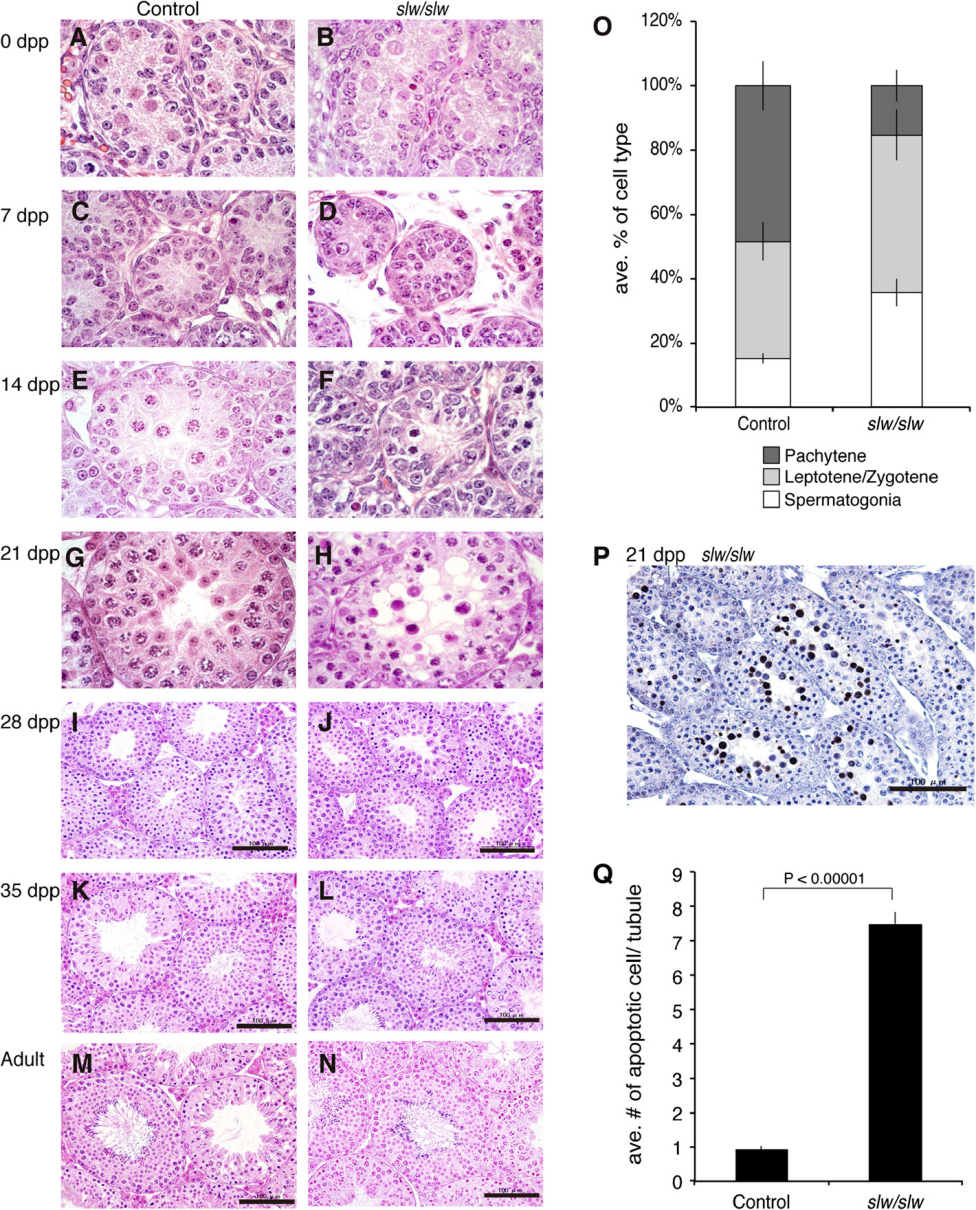
**Histology of testes of *Npr2***^***slw/slw ***^**mice. (A-N)** Hematoxylin and eosin-stained sections of testes of control (left low) and *Npr2*^*slw/slw*^ mice (right low). For histological analysis, n = 5 mice at 0 dpp n = 5 mice at 7 dpp, n = 3 mice at 14 dpp, n = 4 mice at 21 dpp, and n = 1 mouse at 28 dpp, n = 1 mouse at 35 dpp and n = 1 mouse at adult (n = 3, as after weaning mice all combined). At least 10 sections were observed for each testis. Original magnification was x1000 **(A, B, C, D, E, F, G, and H)** and x200 **(I, J, K, L, M, and N)**. Bar indicates 100 μm **(I, J, K, L, M, and N)**. **(O)** Delay of spermatogenic onset in *Npr2*^*slw/slw*^ mice. Cumulative bar graph depicting the percent of the most advanced cell types in seminiferous tubules over a whole testis cross-section was compared between control and *Npr2*^*slw/slw*^ testes at 14 dpp. N = 3 different mice of cross sections were observed for examine progression of spermatogenesis. Total number of tubules observed are 779 and 519 for control and *Npr2*^*slw/slw*^, respectively. The data shown are means (cumulative bar) and SDs (bars). **(P and Q)** Increased cell death in juvenile testes of *Npr2*^*slw/slw*^ mice. TUNEL assay of *Npr2*^*slw/slw*^ testes at 21 dpp **(P)**. Original magnification x200. Bar indicates 100 μm. Comparison of apoptotic cells in the seminiferous tubules of control and *Npr2*^*slw/slw*^ testes at 21 dpp **(Q)**. N = 4 different mice of cross sections were observed for counting apoptotic cells. Total number of tubules observed are 799 and 502 for control and *Npr2*^*slw/slw*^, respectively. The data shown are the means (columns) and SEs (bars).

Histological analyses of testes of *Npr2*^*slw/slw*^ mice revealed no significant differences in overall testis appearance between the control and mutant mice at 0 and 7 dpp (Figure 2A, B, C, D). At 14 dpp, there was evidence of delay in onset and progress of spermatogenesis in *Npr2*^*slw/slw*^ compared to control mice (Figure 2E, F, and O); additionally vacuolization and presence of eosinophilic cells in the seminiferous epithelium was observed in the *Npr2*^*slw/slw*^ testes, but not in control testes, at 21 dpp (Figure 2G, H). At 28 dpp, *Npr2*^*slw/slw*^ mouse exhibited seemingly normal spermatogenesis, however the spermatogenic cells were apparently less advanced compared to control. At this age, elongated spermatids were present in control testes, but not observed in the *Npr2*^*slw/slw*^ testes (Figure 2I and J), indicating delay of spermatogenic progression. However, by 35 dpp, and in adults, the *Npr2*^*slw/slw*^ mice exhibited normal spermatogenesis and germ-cell composition; spermatozoa were present in the lumens of mutant seminiferous tubules (Figure 2L and N) as well as those from control mice (Figure 2K and M).

### Apoptosis of *Npr2* mutant spermatocytes

A TUNEL assay, performed to detect cell death, revealed that apoptotic cells were abundant in late pachytene spermatocytes in *Npr2*^*slw/slw*^ testes at 21 dpp (Figure 2P). The number of apoptotic cell in *Npr2*^*slw/slw*^ at 21 dpp was significantly increased compared to that of control (Figure 2Q).

### Paraphimosis in *Npr2* mutants

**Figure 3:**
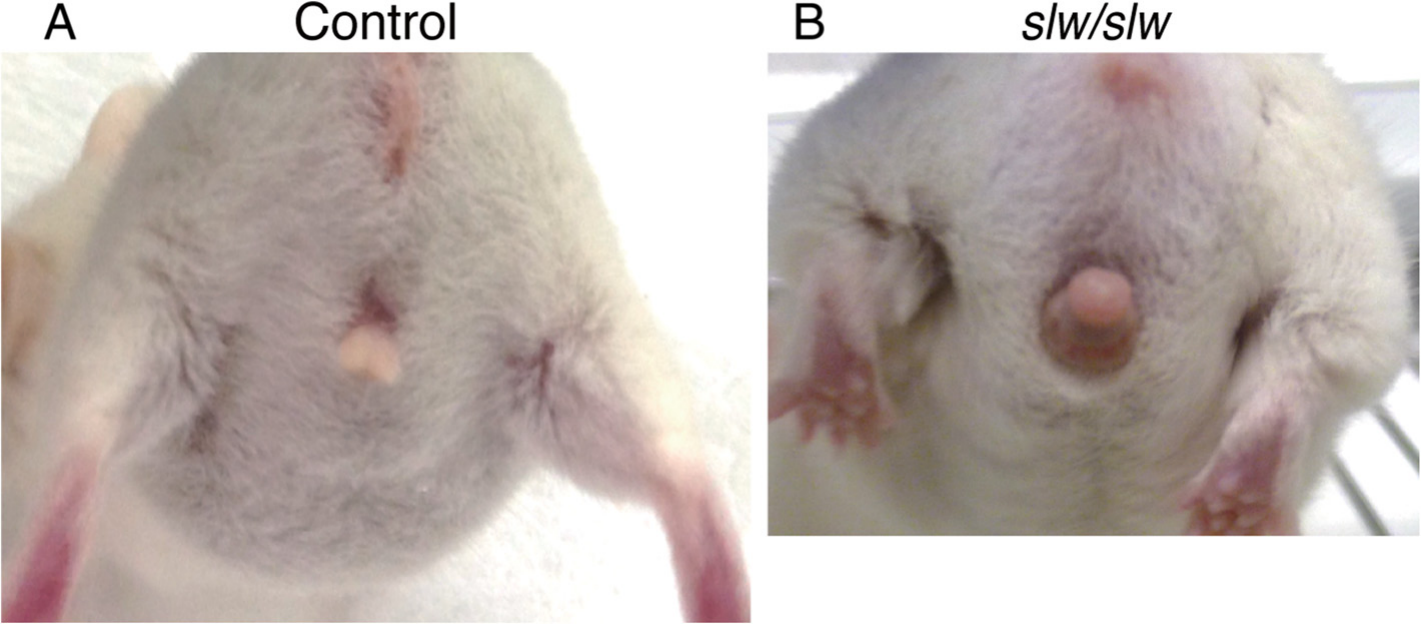
**Paraphimosis in the adult *Npr2***^***slw/slw***^**.** Paraphimosis in the adult *Npr2*^*slw/slw*^ mouse **(B)**. N = >10 and a control mouse from a different litter **(A)**. N = >100.

Anatomically, adult *Npr2*^*slw/slw*^ mice exhibited paraphimosis (erectile dysfunction (ED) accompanying a trapped foreskin), while control adult mice did not (Figure 3A and B). Although the age at which mutant males first exhibited paraphimosis varied among mutant individuals, all adult *Npr2*^*slw/slw*^ mice exhibited paraphimosis. Interestingly, this was a temporary condition; the paraphimosis of *Npr2*^*slw/slw*^ mice did not persist more than one day, although it occasionally recurred. Interestingly, males of another *Npr2* mutant strain, *Npr2*^*cn-2J/cn-2J*^ mice, persisted (data not shown).

## Discussion

This study provides the first description of the male reproductive phenotype of *Npr2*-deficient mice. We found that the developmental onset and acquisition of spermatogenic function is delayed in *Npr2*^*slw/slw*^ mutant mice, but that within 5 weeks, there is restoration of apparently normal spermatogenesis. This developmental delay of spermatogenesis suggests that CNP/NPR-B signaling may play a role in initiation of spermatogenic differentiation. However, the mutant mice did not exhibit any apparent mitotic proliferation abnormality as indicated by PCNA staining (data not shown), and as adults, the *Npr2*^*slw/slw*^ mice exhibited normal germ-cell populations. These results are consistent with the previously reported phenotypes of other *Npr2* alleles, namely the *Npr2*-KO [10] and *Npr2*^*pwe*^ mouse models [9]. Thus we conclude that although CNP/NPR-B signaling is essential for oocyte meiotic arrest in the female mouse [7,17], CNP/NPR-B signaling has different roles in males because spermatogenesis did not suffer meiotic arrest, and thus CNP/NPR-B signaling is not essential for mouse spermatogenesis.

What then might cause the infertility of males bearing homozygous *Npr2* mutations? Possibly malnutrition could play a role. Our observations suggest that abnormalities of testis structure (e.g., vacuolization) and delays in onset of spermatogenic function are more prevalent in mice exhibiting the severe GI disorder characteristic of the *Npr2*^*slw/slw*^ mice. In contrast, both *Npr2*^*slw/slw*^ pups with a latent GI phenotype and *Npr2*^*cn-2J/cn-2J*^ mice, which have no apparent GI disorder, showed normal spermatogenic progression (data not shown). Indeed, *Npr2*^*slw/slw*^ mice surviving to adulthood, though rare, exhibited dwarfism and appeared to be healthy, with apparently normal spermatogenesis. Thus we surmise that severe malnutrition from GI abnormalities may cause the spermatogenic delay at 14 dpp and cell death at 21dpp in *Npr2*^*slw/slw*^ mice. Why the *Npr2*^*cn-2J/cn-2J*^ mice do not suffer from these GI problems is not clear, but may depend on the respective genetic backgrounds. These conclusions about the importance of nutrition are consistent with reports on the role of nutrition in growth and development of the reproductive system [16]; moreover, dietary restriction is known to impact negatively on testicular volume, weight and function [18,19].

There may also be other explanations for the infertility of *Npr2* mutant males other than physical limitations due to dwarfism. The rare *Npr2*^*slw/slw*^ males that survive to adulthood exhibited unique and abnormal penile phenotypes, including paraphimosis and priapism [10]; this unusual phenotype was also found in adult *Npr2*^*cn-2J/cn-2J*^ and *Npr2*-KO mice (data not shown). Sildenafil, commonly known as Viagra® (Pfizer), a specific PDE5 inhibitor that increases concentration of cGMP by nitric oxide (NO) via soluble guanylyl cyclase (sGC) [20], has been used as a medical treatment for ED. Because CNP/NPR-B increases cGMP as well as NO/sGC signaling, the phenotypes of *Npr2*-deficient mice suggest that increase and/or maintenance of cGMP is required for normal erectile function. Our previous report showed that CNP/NPR-B signaling has a critical role in smooth muscle (SM) motility in the gastrointestinal tract [6,14], and therefore prolonged erection in *Npr2*^*slw/slw*^ males is possibly due to abnormal function of the SM of the trabeculae corporum cavernosorum and inability to retain blood flow by contraction due to loss of cGMP production.

## Conclusions

In summary, the present study suggests that NPR-B-mediated CNP signaling may play role in male reproductive function, but CNP/NPR-B signaling is not crucial for spermatogenesis. The sub-fertility in adult *Npr2*^*slw/slw*^ males may instead be caused by penile SM dysfunction. Future studies on CNP will provide information to understand the roles of NPR-B-mediated CNP signaling in spermatogenesis and contribute to the development of novel therapeutics for treatment of male infertility syndromes involving ED.

## Abbreviations

CNP: C-type natriuretic peptide; NPR-B: Natriuretic peptide receptor B; cGMP: Cyclic guanosine monophosphate; PDE5: cGMP-specific phosphodiesterase type 5; GI: Gastrointestinal; SLW: Short-limbed dwarfism; BTB: Blood-Testis barrier; dpp: Day postpartum; NO: Nitric oxide; sGC: Soluble guanylyl cyclase; SM: Smooth muscle; ED: Erectile dysfunction. Nomenclature for CNP and NPR-B is based on the IUPHAR pharmacology database.

## Competing interests

The authors declare that they have no competing interests.

## Authors’ contribution

CS conceived the study, performed histological experiments, was responsible for data analysis and drafted the manuscript. YF was responsible for sampling and data analysis. ST participated in designing the study and introducing the SLW mice. YI, YY and TK participated in introducing SLW mice. TK co-supervised the study and provided SLW mice. TS supervised the study. All authors read and approved the final manuscript.
